# Motor Activity Improves Temporal Expectancy

**DOI:** 10.1371/journal.pone.0119187

**Published:** 2015-03-25

**Authors:** Lilian Fautrelle, Denis Mareschal, Robert French, Caspar Addyman, Elizabeth Thomas

**Affiliations:** 1 Unité de Formation et de Recherche en Sciences et Techniques des Activités Physiques et Sportives, EA2931 Centre de Recherches sur le Sport et le Mouvement, Université Paris Ouest, Nanterre La Défense, France; 2 Centre for Brain and Cognitive Development, Department of Psychological Sciences, Birkbeck University of London, London, United Kingdom; 3 Institut National de la Santé et de la Recherche Médicale, Unité 1093, Cognition, Action et Plasticité Sensori-Motrice, Université de Bourgogne, Dijon, Campus Universitaire, Unité de Formation et de Recherche en Sciences et Techniques des Activités Physiques et Sportives, Dijon, France; 4 Centre National de la Recherche Scientifique, UMR 5022, Laboratoire d’Etude de l’Apprentissage et du Développement, Dijon, France

## Abstract

Certain brain areas involved in interval timing are also important in motor activity. This raises the possibility that motor activity might influence interval timing. To test this hypothesis, we assessed interval timing in healthy adults following different types of training. The pre- and post-training tasks consisted of a button press in response to the presentation of a rhythmic visual stimulus. Alterations in temporal expectancy were evaluated by measuring response times. Training consisted of responding to the visual presentation of regularly appearing stimuli by either: (1) pointing with a whole-body movement, (2) pointing only with the arm, (3) imagining pointing with a whole-body movement, (4) simply watching the stimulus presentation, (5) pointing with a whole-body movement in response to a target that appeared at irregular intervals (6) reading a newspaper. Participants performing a motor activity in response to the regular target showed significant improvements in judgment times compared to individuals with no associated motor activity. Individuals who only *imagined* pointing with a whole-body movement also showed significant improvements. No improvements were observed in the group that trained with a motor response to an irregular stimulus, hence eliminating the explanation that the improved temporal expectations of the other motor training groups was purely due to an improved motor capacity to press the response button. All groups performed a secondary task equally well, hence indicating that our results could not simply be attributed to differences in attention between the groups. Our results show that motor activity, even when it does not play a causal or corrective role, can lead to improved interval timing judgments.

## Introduction

This article investigates how interval timing might be affected by motor activity. Interval timing involves making temporal duration judgments in the range of 500ms to several minutes [1][2][3][4]. In contrast to precision timing, which is involved in many of our automatic motor acts, interval timing requires cognitive resources, and is typically assessed through the use of explicit judgments about interval magnitudes.

Several studies have shown that the accuracy of interval timing judgments can be altered by factors such as cognitive load and stimulus characteristics (see [5] or [3] for reviews). For example, higher stimulus intensity dilates the sense of time [6]. The same is true of stimulus flicker frequency [7]. Stimulus movement and the velocity of this movement also influence interval timing judgments [8][9][10]. Brown [8] demonstrated that a moving stimulus perceived for a fixed amount of time is perceived as being of longer duration than a stationary stimulus perceived for the same amount of time. The author also demonstrated that faster speeds increased perceived time to a greater degree than slower speeds. Tomassini et al. [10] extended these results to show that they held not only for visual but tactile stimuli as well. The position of the stimulus in a repetitive sequence of the stimulus also plays a role in our perception of its duration. The first stimulus is judged to last longer than the other stimuli in the sequence [11][12].

Another factor found to influence estimates of interval duration, has been attention and cognitive load [12][13][14][15][16]. Block, Hancock, and Zakay [16] after having analyzed the results from a total of 117 studies found a striking interaction between the type of time judgment requested and cognitive load. High cognitive load increases your estimates in the case of retrospective timing, whereas high cognitive load decreases your estimates in the case of prospective timing.

In contrast to the above studies, however, very few investigations have explicitly attempted to explore how motor activity can affect interval timing. A notable exception is the work of Haggard et al. [17], who found that subjects systematically reported that the interval between a button press and the resulting stimulus onset was shorter than its actual duration. In other words, the motor act (i.e., pressing the button) led to an underestimation of the time between the button press and the stimulus onset. This compression of the time interval between the motor act and the appearance of the stimulus can even lead to a reversal in the judgment of which event occurred first [18]. It should be noted, however, that the button press in these studies plays a ‘causal’ role i.e. stimulus onset is caused by the button press. This is in contrast to our study where the participants press a button in response to a stimulus appearance. Other more indirect signs of the influence of the motor domain come from studies that show that head position can influence timing judgments [19] or that patients with neuromuscular disorders such as dystonia [20] or Parkinson’s disease [21] also display inaccurate temporal judgments.

In the current investigation, we reasoned that if interval timing judgments can be influenced by visual, auditory or tactile sensory input, the same might be true of motor activity. Strong a priori support for this hypothesis comes from the fact that several of the neural structures important in interval timing are also very important in motor activity; especially, the basal ganglia [22][23][24][25] and the supplementary motor cortex [3][4] [26][27][28]. The former structure has been found to be more important in explicit timing tasks while the cortical premotor areas are involved in both explicit and implicit perceptual timing tasks or ‘temporal expectation’ [29]. Given the shared neural substrates for motor activity and interval timing judgments, it is reasonable to ask if motor activity could influence our interval timing judgments. Indeed, some researchers have even suggested that infants develop their sense of timing through motor activity [30][31][32]. Furthermore, if the hypothesis on adult temporal judgments is true, would high-amplitude motor activity have a greater influence on interval timing than low-amplitude motor activity? Stronger muscular contractions require higher motoneuron activity [33]. Penfield & Rasmussen (1952) [34] showed that there is a somatotopic organization in the precentral gyrus of the human brain. Movements involving more than one limb would therefore involve a greater area of the motor cortical map than those involving just one limb. In addition, certain cognitive activities recruit motor areas of the brain without eliciting visible motor activity. Motor imagery is one such activity [35][36]. Thus, if real actions impact on temporal judgments, then this may also be true of imagined actions.

To explore these issues, we carried out an interval timing task in which participants were asked to respond to the presentation of regularly appearing visual stimuli. Reductions in the reaction time to the visual presentations were taken as an indicator of an improved ability to predict the appearance of the next stimulus. Several studies have now shown that response times are reduced when subjects expect the appearance of a stimulus at a particular time (temporal expectancy) [24][29][37][38][39]. To study the effect of motor activity on temporal expectancy, we compared the interval timing performance of groups that trained with or without motor activity. The different training groups were as follows: (1) a group training with a simple motor task (SMT), (2) a group training with a complex motor task (CMT), (3) a group training with motor imagery (MI), (4) a group training with visual presentation only (VI), (5) a group training with a complex motor action but irregular stimulus timings (IS) and finally (6) a group with no training (CRTL). As the areas of the brain involved in motor activity are also important for temporal expectancy, we expected that the groups training on interval timing using motor responses (SMT, CMT, MI) would have a different sense of timing compared to those without any motor activity (VI, CRTL). We expected in addition, that the amplitude of motor activity would have an effect on this modification. In other words, we expected to see significant differences in the gains obtained from the SMT, CMT and MI training. If the changes observed in the groups that had trained with motor activity were actually due to an improved capacity to perform a perceptual task and not just due to an increased ease with performing a motor act, we would also observe differences in the IS group compared to the SMT, CMT or MI group.

## Methods

### Participants

One hundred and twenty healthy participants (68 males and 52 females; mean age = 27.4 years, SD = 5) volunteered for the experiment. All the participants were students and were rewarded with a USB memory stick for their participation in this study. They had normal or corrected-to-normal vision and none had a previous history of neuromuscular or neurological disorder. All the participants were right handed as assessed by the Edinburgh Handedness Inventory [40]. The experiment conformed to the declaration of Helsinki. The study was approved by the Ethics committee of the University of Burgundy. Verbal consent as approved by the ethics committee of the University of Burgundy was obtained from all participants.

Participants in the motor-imagery condition were first screened for their capacity for motor imagery according to previously established protocols [41]. This consisted of asking participants to perform 10 real pointing movements or to imagine the same movements 10 times at a natural speed (the order was randomized). The durations necessary to execute the real and imaginary movements were then compared. A difference of more than 7% between the average durations of the real and the imagined pointing movements led to the exclusion of the subject from the motor-imagery group. Based on this criterion, seven participants were rejected from the study. Among the accepted participants, the biggest difference between the average durations of the real and imagined movement was 0.140 s (i.e., a difference of 5.9%).

### Materials and stimuli

The visual stimuli were projected onto a translucent 2x2 meter screen by a CRT video projector. The spatial resolution of the visual display system was 1024x768 pixels with a vertical refresh sampling rate of 60 Hertz. The visual stimuli consisted of a white dot (0.4 m in diameter) presented for a duration of 0.5 s. on a black background. During the training sessions a random 10% of these dots were green, while the remaining 90% were white. All experiments were conducted with the participants either standing or sitting 2 m. from the screen.

A standard single-button joystick was used. The timings of the visual stimuli and the joystick were synchronized and recorded at a sampling frequency of 5 kHz. The signals were processed with a multichannel analog-to-digital converter (Biopac Systems, Inc., Goleta, California). Response time was defined as the duration between the appearance of the visual stimuli and the moment at which participants pressed the joystick button.

### Procedure

**Fig 1:**
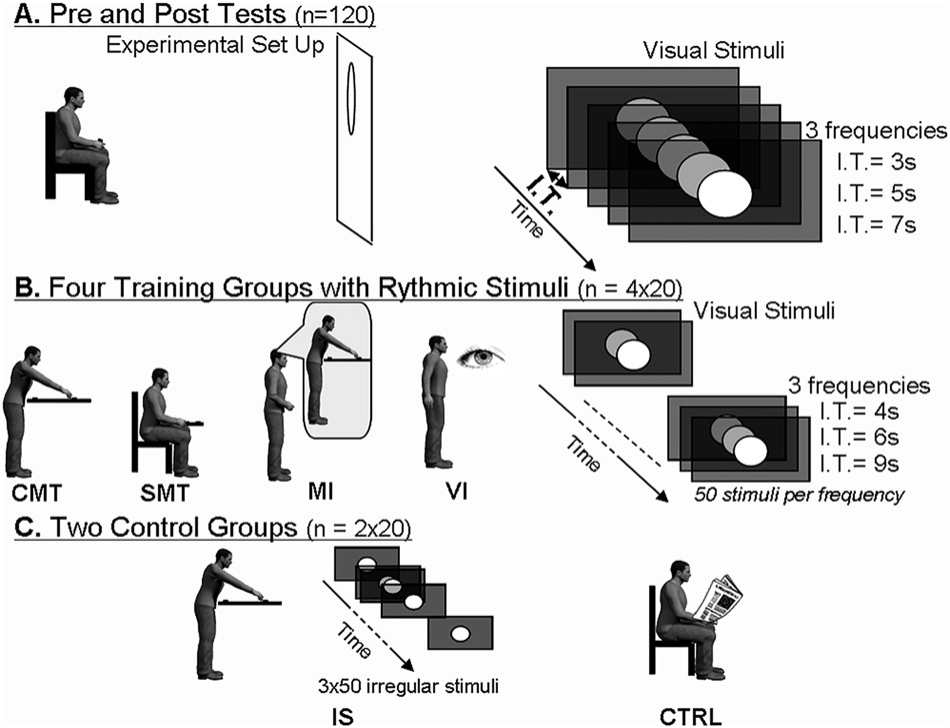
Experimental setup and design.

The full design and procedure are illustrated in Fig. 1. The experiment consisted of a pre-training session (i.e., a "familiarization phase") lasting 10 minutes, during which button-press responses to the regular appearance of the visual target were recorded. This was followed by a training session (approximately 25 minutes) during which the participants were once again presented with regular appearances of the stimulus. However, during training, responses could involve more complex motor tasks such as reaching or leaning over. We describe the different training conditions in more detail below. Finally in the post-training session (10 minutes), exactly the same button-press that had been used in the pre-training task was repeated. This enabled a comparison of the response times before and after the training sessions.

### Pre-training phase

During the pre-training phase, participants sat in a chair located 2 meters in front of the projection screen. They were instructed to respond to the regular presentation of the visual stimulus by pressing their thumb on the button of a joystick held in their right hand. Participants were first presented with three separate sequences consisting of 9 presentations of the white dot occurring at regularly spaced 3-, 5- or 7-second intervals depending on the sequence. They were instructed not to respond to the first three stimulus appearances in each sequence as these were used to familiarize them with the interval duration. The participants were then asked to press the joystick button as soon as they saw the remaining six presentations of the white dot. The order in which the 3-, 5- and 7-second sequences were seen, was appropriately randomized across participants.

### Training phase

Participants were divided into 6 groups of 20 as follows: (1) training with a simple motor task (SMT), (2) training with a complex motor task (CMT), (3) training with motor imagery (MI), (4) training with visual presentation only (VI), (5) training with a complex motor action and irregular stimulus timings (IS) and (6) no training (CRTL). The training phase for each individual, irrespective of the group they belonged to, lasted 25 minutes.

All training groups, with the exception of the IS and CTRL groups, were presented with sequences comprised of 50 regularly spaced appearances of the white-dot described above. The presentations of the stimulus during the training sequences occurred at intervals of 4, 6 or 9 seconds. The order of the sequences was randomized as described above. The response that the participants were required to make upon seeing the white-dot depended on their training group, as described in more detail below. Note that for the CMT and SMT groups, in which specific movements were required, the presence of switches at the beginning and ending point of the movement enabled us to ensure that the full movement had been carried out.

#### (i) The CMT group

Participants stood 2 m. from the screen. On every appearance of the visual stimuli, they had to reach for and touch a button located in the sagittal plane, 90 cm from the starting point in front of them and 15 cm below the xyphoid process. This activity was labeled as a complex motor task because its successful accomplishment required a forward trunk bend and arm movement, while simultaneously maintaining equilibrium.

#### (ii) The SMT group

Participants sat 2 m. from the screen. They responded to the presentation of a visual stimulus by reaching for and pressing a button located in the sagittal plane, 20 cm from a starting point in front of them and 15 cm above the xyphoid process. This activity was labeled as a simple motor task because the target could be reached with the arm alone from a sitting position.

#### (iii) The MI group

The participants’ initial posture was identical to that of the CMT group. However, the participants only had to imagine reaching the target at each appearance of the visual stimulus without moving.

#### (iv) The VI group

Participants sat 2 m. from the screen and were required only to watch the visual stimuli attentively without overtly responding to anything. The participants had their hands on their knees.

#### (v) The IS group

The participants’ initial posture, the pointing apparatus, and the instructions (i.e., to reach for and touch the target at the appearance of the visual stimuli) were exactly the same as in the CMT training group. However the manner in which the visual stimulus was presented differed. One hundred and fifty timing intervals (50x4s, 50x6s, and 50x9s) were randomized and distributed across three sessions of 50 presentations of each of the visual stimulus. Given the random nature of the stimulus presentations, the participants were unable to estimate the durations of the inter-stimulus intervals. The number of stimulus presentations and the sum of the interval durations were the same as those of the three other groups in which a “reach and touch” response was required.

#### (vi) The CTRL group

In this control group participants read a newspaper for 25 minutes.

To ensure equal levels of engagement, participants in all groups (except the CTRL group) took part in a secondary task during training. A random ten percent of the stimulus dots were green and, at the end of the recording sessions, participants were required to report the number of green dots that had appeared throughout the entire training session.

### Post-training phase

All participants repeated the pre-training tests that began the experiment. The difference in their performance during the pre-training phase and the present post-training phase was compared.

## Results

**Fig 2:**
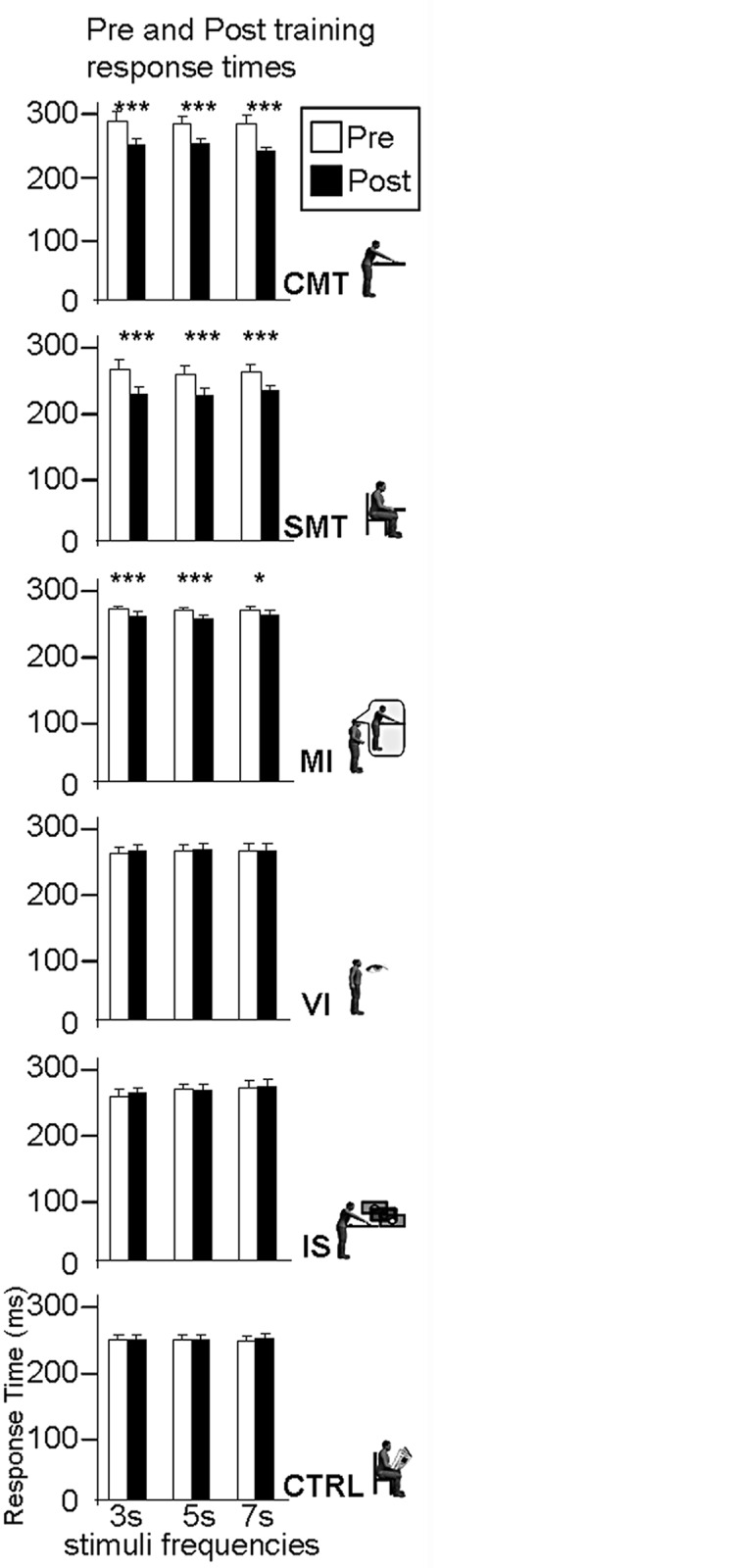
Pre- and post-training response times (mean ± std) for all the participants at every stimulus frequency. The experimental groups tested were from top to bottom, the CMT, SMT, MI, VI, IS, and CTRL.*p<0.05, **p<0.01, ***p<0.001.

The effect of motor training on interval timing was assessed by comparing the post-training phase response times with those obtained in the pre-training phase (Fig. 2). The response times were analyzed with a mixed ANOVA. Session (pre-training, post-training) and Interval Duration (3, 5, 7 s.) were within-participant measures while Training Group (CMT, SMT, MI, IS, VI, CTRL) was a between-participant factor. Tukey HSD post hoc tests were carried out where appropriate.

**Fig 3:**
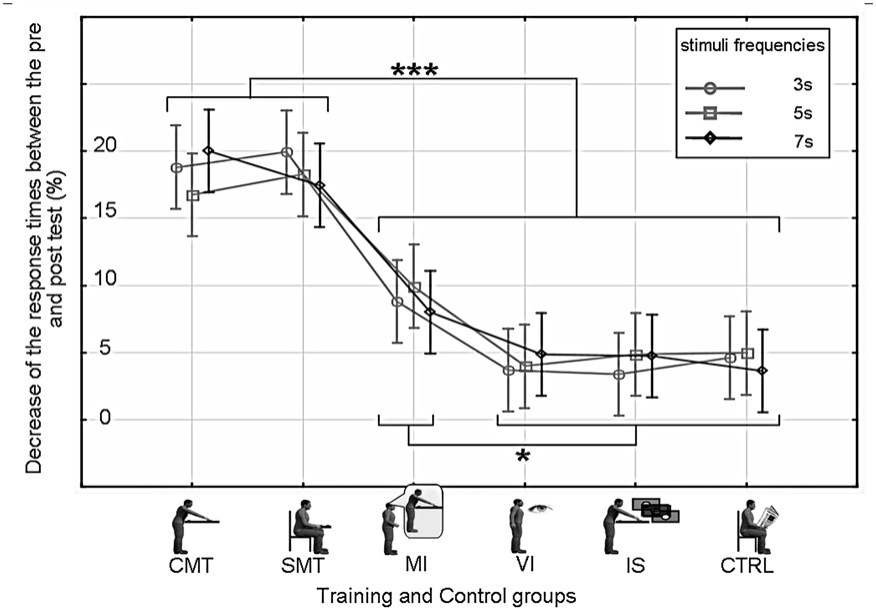
Percentage decrease in the response time (mean ± std) in the post-training test phase when compared to that in the pre-training phase. Gain ratios are reported for all the experimental groups (x-axis) and every test frequency (3s with rounds, 5s with squares and 7s with diamonds). *p<0.05, ***p<0.001

The ANOVA revealed a main effect of Training Group (*F*(5, 594) = 8.3, *p*<0.0001, η^2^ = 0.36) and Session (*F*(1, 594) = 138.6, *p*<0.0001, η^2^ = 0.42), as well as a significant interaction of Training Group x Session (*F*(5, 594) = 45.8, *p*<0.0001, η^2^ = 0.1). There was no main effect of Interval Duration (*F*(2, 1188) = 0.59, *p* = 0.55, η^2^ = 0.01). No significant differences in response times were observed between the different groups in the pre-training phase (p>0.05, Tukey HSD posthoc). The Training Group x Session interaction could be explained by the fact that pre-training and post-training phase response times were only significantly different when motor training or motor imagery was involved (Fig. 2), i.e., for the CMT, the SMT and MI groups (p<0.05, Tukey HSD posthoc). Response times (RT) following training were reduced in these three groups, reflecting an improved temporal expectation for the appearance of the upcoming stimulus. The mean gains were 40 (SD 19) ms, 33 (SD 20) ms and 11 (SD 9) ms for the CMT, SMT and MI groups respectively (Fig. 3). Reductions in response times for the remaining groups were not significant (The mean values had in fact increased by 1ms for the VI and IS groups and decreased by 1ms for the CTRL group).

There were no significant differences between the gains in the CMT and SMT groups (p>0.05, Tukey HSD). However, post-training phase response times for both the SMT and CMT groups were significantly shorter than those in the MI group (p<0.001, Tukey HSD posthoc). Although, the RT improvement was not as great as for the motor training groups, the MI group was also found to have significantly shorter test phase response times than all the non-motor-training groups (p<0.05, Tukey HSD posthoc) (Fig. 3).

Finally, in the visual training (VI), the irregular target (IS), and in the control group (CTRL) there were no significant RT improvements between the pre and post-training phases. The lack of significant RT improvement (p>0.05, Tukey HSD posthoc) observed in the irregular stimulus (IS) group indicates that the gains observed in the CMT, SMT and MI groups were not simply due to a general sensorimotor facilitation associated with making (or imagining) a movement.

All participants had 100% accuracy in the secondary task in all conditions. This indicates that there were no major differences in attention levels between the training groups. No responses prior to the appearance of the stimulus were observed in any of the conditions. This suggests that the RT improvements observed in the test phase were not simply due to a systematic (and intentional) underestimation of when the stimulus was going to appear.

## Discussion

These experiments indicate that repetitive, time locked motor activity is able to improve temporal expectancy. Groups that had incorporated motor activity into their training protocol with regularly spaced stimuli showed shorter post-training phase response times than the group with only visual training. The shorter response times suggest better preparation and more accurate anticipation of the appearance of the dot on the screen (i.e., improved implicit interval timing). Since we did not find any significant differences between the pre-training and post-training phase response times for those who received no training at all (CTRL), the performance improvement in the motor and motor-imagery training groups can be attributed specifically to the training phase. This raises the questions of how and why?

Could the improved performance of the motor training and motor-imagery groups be due to differences in attention or vigilance in these groups? To rule this out, we added a secondary task to the basic interval timing task. This task involved asking participants to count the number of green colored targets that appeared during the training sequences. Performance on this secondary task was constant and high across all training conditions, suggesting that there were no significant differences in the level of alertness across the different groups.

Another possible explanation for the improvement in reaction times observed in the motor training groups is some kind of general “sensorimotor facilitation” for performing a motor act. We use the word ‘general’ here because the motor tasks used in the training were quite different from those used in the test phase. Specifically, testing involved pushing a button on a joy stick with the thumb, whereas motor training involved whole arm or body pointing towards a target. However, this possibility was also ruled out by a lack of significant improvements in response times in the IS group. This group performed the same movement as those in the CMT group, but with an irregular unpredictable stimulus. Moreover, the total number of movements in this group was the same as in the motor-training groups with a regular stimulus. If the improvements obtained with the other motor training groups had been just due to an improvement of the speed of the motor act itself, without any perceptual anticipation, we should also have seen an improvement in the response times of the IS group, which was not the case.

One interesting observation is that the response-time improvements obtained in the simple motor activity (SMT) group were not significantly different from those in the complex motor training (CMT) group. This may lead one to make the conclusion that movement amplitude does not play a role in temporal expectancy. This is probably not accurate however, since as we explain in the paragraph below, the reduction in temporal expectancy with the MI group was significantly less than for the SMT or CMT groups (Fig. 3). Motor imagery involves the activation of motor area, but at a lower amplitude than what is observed during real movement [42]. Since temporal expectancy is not significantly different between the SMT and CMT groups, but is significantly lower in the MI group (Fig. 3), this indicates a nonlinear dependence on the amplitude of activation in the motor areas. A previous study by Gavazzi et al [43] has also shown how temporal judgments can change based on the specific motor act that is used to reproduce the stimulus duration.

The small, but significant performance improvement that was observed in the motor-imagery group suggests that simply activating the mental and neural structures necessary to support movement, but without an actual movement is sufficient to produce improved accuracy in interval timing. These results also help to rule out the possibility that simple repetitive haptic stimulation is responsible for the observed improvements.

One might be a little surprised by the lack of any significant reduction in response times for the group that only underwent visual training (VI group). Meegan et al [44] reported that participants who had taken part in perceptual training also showed improvements in a motor task involving interval timing. The reason for the difference in our results may lie primarily in the type of stimulus that was used. Meegan et al [44] had used an auditory stimulus while the sensory stimulus in our study was visual. A study by Cicchini et al [45] would also seem to corroborate this explanation. These authors found that more accurate interval judgments were made when auditory tones rather than visual stimuli were used.

Any mechanisms proposed to explain how motor activity could improve temporal expectancy would have to be of a non-specific nature. This is because both the temporal spacing of the stimuli and the responses to them were different in the training and the test phases. Specifically, the intervals to be timed in the test phase (3, 5, and 7s) were not the same as those used in the training phase (4, 6, and 9s). And, more importantly, the motor activity used in the testing phase was very different from those used in the training phase.

One possible explanation for our findings may lie in what Eagleman [5] proposed when attempting to explain why the first interval in a sequence appears to last longer than subsequent ones. This shortening of perceived durations of successive stimuli in comparison to the first, have now been observed by several researchers [5][11][12][46][47]. Eagleman [5] suggests that this phenomenon is due to the well-known observation of decreased neural firing in the brain following repeated stimulation due to neural adaptation. Motor training may increase the general level of activity in areas that are also implicated in interval timing. This may then reduce the effects of repeated stimulations. Such an explanation would hold both for models that ascribe timekeeping to a dedicated neural pacemaker structure [48][49][50][51] as well as for models that attribute timing to a more distributed spatiotemporal neuronal map [1][2][32][52]. Of course, if this were true, then one might ask why no response-time improvements were observed in the irregular stimulus (IS) condition. One answer is that the habituation produced in the IS condition with an irregular stimulus may have been significantly less than in the conditions with a temporally predictable stimulus.

Another possible explanation for the facilitatory effect of motor activity on interval timing is that the repeated pairing of the two leads to heterosynaptic Hebbian association [53] and, as a result, some motor neurons are recruited for interval timing. This would then allow for the combination of cues from two different modalities, the visual and the motor, hence improving the final interval estimation that has to be made. It has been suggested that the brain uses a Bayesian framework to integrate multiple sensory cues [54][55][56][57]. In this study we suggest that not only sensory input, but also input from the motor system may contribute to improving perceptual and cognitive estimates. It should also be noted that motor activity not only provides the signals for the execution of a movement but also generates ‘sensory’ information on the movement in the form of the efference copy [58][59][60]. As Hebbian association are based on the temporal proximity of two signals [61][62][63], the spatial proximity of the motor and interval timing systems would facilitate an association of the two.

The current study opens up several questions. If the improved interval estimation was obtained through the combination of stimuli from different modalities, it is possible that associating an auditory stimulus with the presentation of the white dots in our experiment may also have reduced reaction times. Indeed several studies have now shown that temporal estimation relies more heavily on auditory than visual information [64][65][66]. Future comparative studies will have to be carried out in order to determine if associating an auditory tone with the presentation of the white dots would improve interval timing estimates more than the association with motor activity.

Finally, it should be noted that the motor task used in the training did not achieve its effects through corrective feedback. An example of this would be catching a ball. In this case, a failed catch would indicate among other things, a failed interval time judgment. The error feedback can then be used to re-calibrate the timing system. Several researchers have shown that the re-calibration of interval timing judgments based on prior errors are in agreement with the predictions of Bayesian integration [67][68]. While motor activity clearly has this potential, it did not play such a role in our study. All motor activity during training took place *after* stimulus presentation, and, therefore, could not have provided error feedback to adjust timing judgments. This particular aspect sets our study apart from the Haggard investigation [17].

Future studies will be needed to verify if the effects that we have observed with motor activity also hold for other protocols of interval timing judgments. For example, would temporal expectancy also be improved if we used filled intervals for the stimulus? Other than the implicit tasks that were used in this investigation, future studies should also involve more explicit timing tasks such as the reproductions of temporal intervals, comparisons of time intervals or the detection of rhythmic or irregular sequences. Finally the timing performance improvements observed when using motor activity must be compared with those that might be obtained by associating other sensory stimuli—for example, auditory stimulation—with interval timing judgments. This would help us to determine whether the activation of the motor areas provides specific, additional benefits compared to the activation of other sensory areas during interval timing.

In conclusion, our study demonstrates that repetitive, time locked motor activity is able to improve temporal expectancy. This is the case even when the motor activity is non-causal with respect to the stimulus appearance and does not provide error feedback that could be used to improve the interval timing performance.

## Supporting Information

S1 Dataset Data for all the groups and all the trials. (XLS)

## References

[1] Ivry RB, Schlerf JE (2008) Dedicated and intrinsic models of time perception. Trends in Cognitive Sciences 12: 273–280. 10.1016/j.tics.2008.04.002 18539519 PMC4335014

[2] Buonomano D, Laje R (2010) Population clocks: motor timing with neural dynamics. Trends in Cognitive Science 14: 520–527. 10.1016/j.tics.2010.09.002 20889368 PMC2991437

[3] Grondin S (2010) Timing and time perception: a review of recent behavioral and neuroscience findings and theoretical directions. Attention, Perception and Psychophysics 72: 561–82. 10.3758/APP.72.3.561 20348562

[4] Coull JT, Cheng RK, Meck WH (2011) Neuroanatomical and neurochemical substrates of timing. Neuropsychopharmacology 36: 3–25. 10.1038/npp.2010.113 20668434 PMC3055517

[5] Eagleman DM (2008) Human time perception and its illusions. Current Opinion in Neurobiology 18: 131–6. 10.1016/j.conb.2008.06.002 18639634 PMC2866156

[6] Xuan B, Zhang D, He S, Chen X (2007) Larger stimuli are judged to last longer. Journal of Vision 7:1–5. 10.1167/7.10.2 17997671

[7] Kanai P, Paffen CL, Hogendoorn H, Versraten FA (2006a) Time dilation in dynamic visual display. Journal of Vision 6: 1421–1430. 17209745 10.1167/6.12.8

[8] Brown SW (1995) Time, change and motion: the effects of stimulus movement on temporal perception. Perception & Psychophysics 57: 105–116. 7885802 10.3758/bf03211853

[9] Kaneko S, Murakami I (2009) Perceived duration of visual motion increases with speed. Journal of Vision 9: 14 10.1167/9.13.14 19761329

[10] Tomassini A, Gori M, Burr D, Sandini G, Morrone MC (2011) Perceived duration of visual and tactile stimuli depends on perceived speed. Frontiers in Integrative Neuroscience 5: 51 10.3389/fnint.2011.00051 21941471 PMC3170919

[11] Pariyadh V, Eagleman DM (2007) The effect of predictability on subjective duration. PLOS One 2:1264. 10.1371/journal.pone.0001264 PMC2082074 18043760

[12] Rose D, Summers J (1995) Duration illusions in a train of visual stimuli. Perception, 24: 1177–1187. 8577576 10.1068/p241177

[13] Brown SW, Boltz M (2002) Attentional processes in time perception: Effects of mental workload and event structure. Journal of Experimental Psychology: Human Perception and Performance, 28: 600–615. 12075891

[14] Buhusi CV, Meck WH (2005) What makes us tick? Functional and neural mechanisms of interval timing. Nature Reviews Neuroscience 6: 755–765. 16163383 10.1038/nrn1764

[15] Burle B, Casini L (2001) Dissociation between activation and attention effects in time estimation: Implications for internal clock models. Journal of Experimental Psychology: Human Perception and Performance 27: 195–205. 11248933 10.1037//0096-1523.27.1.195

[16] Block RA, Hancock PA, Zakay D (2010) How cognitive load affects duration judgments: A meta-analytic review. Acta Psychologica 134:330–343. 10.1016/j.actpsy.2010.03.006 20403583

[17] Haggard P, Clark S, Kalogeras J (2002) Voluntary action and conscious awareness. Nature Neuroscience 5: 382–5. 11896397 10.1038/nn827

[18] Stetson C, Cui X, Montague PR, Eagleman DM (2006) Motor-sensory recalibration leads to an illusory reversal of action and sensation. Neuron 51: 651–659. 16950162 10.1016/j.neuron.2006.08.006

[19] Vicario CM, Martino D, Pavone EF, Fuggetta G (2011) Lateral head turning affects temporal memory. Percept Mot Skills 113: 3–10. 21987905 10.2466/04.22.PMS.113.4.3-10

[20] Avanzino L, Martino D, Martino I, Pelosin E, Vicario CM, Bove M, et al (2013) Temporal expectation in focal hand dystonia. Brain 136: 444–454. 10.1093/brain/aws328 23361064

[21] Allman M, Meck WH (2012) Pathophysiological distortions in time perception and timed performance. Brain 135: 656–677. 10.1093/brain/awr210 21921020 PMC3491636

[22] Artieda J, Pastor MA, Lacruz F, Obeso JA (1992) Temporal discrimination is abnormal in Parkinson’s disease. Brain 115: 199–210. 1559154 10.1093/brain/115.1.199

[23] Meck WH (2005) Neuropsychology of timing and time perception. Brain & Cognition, 58, 1–8. 15878722 10.1016/j.bandc.2004.09.004

[24] Coull JT, Nobre AC (1998) Where and when to pay attention: The neural systems for directing attention to spatial locations and to time intervals as revealed by both PET and fMRI. J Neuroscience 18:7426–7435. 9736662 10.1523/JNEUROSCI.18-18-07426.1998 PMC6793260

[25] Bueti D, Walsh V, Frith C, Reese G (2008) Different brain circuits underlie motor and perceptual representations of temporal intervals. J Cognitive Neurosci 20: 204–214. 10.1162/jocn.2008.20017 18275329

[26] Mita A, Mushiake H, Shima K, Matsuzaka Y, Tanji J (2009) Interval time coding by neurons in the presupplementary and supplementary motor areas. Nature Neuroscience 12: 502–7. 10.1038/nn.2272 19252498

[27] Schwartz M, Rothermich K, Kotz SA (2012) Functional dissociation of pre-SMA and SMA-proper in temporal processing. Neuroimage 60: 290–8. 10.1016/j.neuroimage.2011.11.089 22178297

[28] Macar F, Coull J, Vidal F (2006) The supplementary motor area in motor and perceptual time processing: fMRi studies. Cognitive Processing 7:89–94. 16683171 10.1007/s10339-005-0025-7

[29] Coull JT, Nobre AC (2008) Dissociating explicit timing from temporal expectation with fMRI. Current Opinion in Neurobiology 18: 137–144. 10.1016/j.conb.2008.07.011 18692573

[30] Droit-Volet S (1998) Time estimation in young children: an initial force rule governing time production, J Exp Child Psychol. 68: 236–249. 9514772 10.1006/jecp.1997.2430

[31] Michon JA, Pouthas V, Jackson JL (1989) Guyau and the idea of time. Elsevier, Amsterdam.

[32] Addyman C, French RM, Mareschal D, Thomas E (2011). Learning to perceive time: A connectionist, memory-decay model of the development of interval timing in infants In Proceedings of the Thirty-Third Annual Cognitive Science Society Conference. Carlson L, Holscher C and Shipley T (Eds), Austin TX, p 354–359.

[33] Latash M (2008) Neurophysiological basis of movement p 53 Human Kinetics. Champaign, Illinois.

[34] Penfield W, Rasmussen T (1952) The Cerebral Cortex of Man. New York Macmillan.

[35] Gao Q, Duan X, Chen H (2011) Evaluation of effective connectivity of motor areas during motor imagery and execution using conditional Granger causality. Neuroimage 15: 1280–8. 10.1016/j.neuroimage.2010.08.071 20828626

[36] Szameitat AJ, McNamara A, Shen S, Sterr A (2012) Neural activation and functional connectivity during motor imagery of bimanual everyday actions. PLoS One 7: e38506 10.1371/journal.pone.0038506 22701655 PMC3368848

[37] Nobre AC (2001) Orienting attention to instants in time. Neuropsychologia 39: 1317–1328. 11566314 10.1016/s0028-3932(01)00120-8

[38] Correa A, Lupianez J, Milliken B, Tudela P (2004) Endogenous temporal orienting of attention in detection and discrimination tasks. Perception and Psychophysics 66:264–278. 15129748 10.3758/bf03194878

[39] Correa A, Lupianez J, Tudela P (2005) Attentional preparation based on temporal expectancy modulates processing at the perceptual level. Psychonomic Bulletin & Reviews 12: 328–334. 10.3758/bf03196380 16082814

[40] Oldfield RC (1971) The assessment and analysis of handedness: the Edinburgh inventory. Neuropsychologia 9: 97–113. 5146491 10.1016/0028-3932(71)90067-4

[41] Papaxanthis C, Pozzo T, Skoura X, Schieppati M (2002) Does order and timing in performance of imagined and actual movements affect the motor imagery process? The duration of walking and writing task. Behav Brain Res 134:209–15. 12191807 10.1016/s0166-4328(02)00030-x

[42] Porro CA, Francescato MP, Cettolo V, Diamond ME, Baraldi P, Zuiani C, et al (1996) Primary Motor and sensory cortex activation during motor performance and motor imagery: A functional magnetic resonance imaging study. J. Neurosci. 16: 7688–7698. 8922425 10.1523/JNEUROSCI.16-23-07688.1996 PMC6579073

[43] Gavazzi G, Bisio A, Pozzo T (2013) The perception of visual motion is tuned by the motor representation of human actions. Scientific Reports 3:1–8. 10.1038/srep01168 PMC3558721 23378903

[44] Meegan DV, Aslin RN, Jacobs RA (2000) Motor timing learned without motor training. Nature Neuroscience 3: 860–862. 10966614 10.1038/78757

[45] Cicchini GM, Arrighi R, Cecchiti L, Guisti M, Burr D (2012) Optimal encoding of interval timing in expert percussionists. J. Neuroscience 32: 1056–1060. 10.1523/JNEUROSCI.3411-11.2012 PMC6621155 22262903

[46] Hodinott-Hill I, Thilo KV, Cowey A, Walsh V (2002) Auditory chronostasis: Hanging on the telephone. Curr Biol 12: 1779–1781. 12401174 10.1016/s0960-9822(02)01219-8

[47] Kanai R, Watanabe M (2006b) Visual onset expands subjective time. Percept Psychophys 68: 1113–1123. 17355036 10.3758/bf03193714

[48] Gibbon J (1977) Scalar expectancy theory and Weber’s law in animal timing. Psychological Review 84: 279–325.

[49] Treisman M, Faulkner A, Nash PLN, Brogan D (1990) The internal clock: Evidence for a temporal oscillation underlying time perception with some estimates of its characteristic frequency. Perception 19: 705–743. 2130371 10.1068/p190705

[50] Wearden JH (2003) Applying the scalar timing model to human time psychology: Progress and challenges In Helfrich H (Ed), Time and mind II (pp 21–39). Gottingen: Hogrefe & Huber.

[51] Rammsayer TH, Ulrich R (2001) Counting models of temporal discrimination. Psychonomics Bulletin and Review 8: 270–277. 11495114 10.3758/bf03196161

[52] French RM, Addyman C, Mareschal D, Thomas E (2014) GAMIT: A Fading Gaussian Activation model of interval timing. Timing and Time Perception Reviews 1:1–17

[53] Bailey CH, Giustetto M, Huang YY, Hawkins RD, Kandel ER (2000) Is heterosynaptic modulation essential for stabilizing Hebbian plasticity and memory? Nature Review Neuroscience 1:11–20. 11252764 10.1038/35036191

[54] Pouget A, Deneve S, Duhamel JR (2002) A computational perspective on the neural basis of multisensory spatial representations. Nature Rev. Neuroscience 3: 741–747. 12209122 10.1038/nrn914

[55] Kording KP, Wolpert DM (2004). Bayesian integration in sensorimotor learning. Nature 427:244–247. 14724638 10.1038/nature02169

[56] Wolpert D (2007) Probabilistic models in human sensorimotor control. Human Movement Science 26:511–524. 17628731 10.1016/j.humov.2007.05.005 PMC2637437

[57] Shi Z, Church RM, Meck W (2013) Bayesian optimization of time perception. Trends in Cognitive Sciences 17: 556–64. 10.1016/j.tics.2013.09.009 24139486

[58] Holst E, von Mittelstaedt H (1950) Das Reafferenzprinzip. Wechselwirkungen zwischen Zentralnervensystem und Peripherie. Naturwissenschaften 37: 464–476.

[59] Wolpert DM, Kawato M (1998) Internal models of the cerebellum. Trends in Cognitive Science 2: 338–347. 21227230 10.1016/s1364-6613(98)01221-2

[60] Wurtz R, Joiner WM, Bermann RA (2011) Neuronal mechanisms for visual stability: progress and problems. Phil Trans R Soc 366: 492–503. 10.1098/rstb.2010.0186 21242138 PMC3030829

[61] Bliss TVP, Lomo T (1973) Long lasting potentiation of synaptic transmission in the dentate area of the anesthetized rabbit following stimulation of the perforant path. J Physiol 232: 331–356. 4727084 10.1113/jphysiol.1973.sp010273 PMC1350458

[62] Nicoll RA, Kauer JA, Malenka RC (1988) The current excitement in long term potentiation. Neuron 1: 97–103. 2856092 10.1016/0896-6273(88)90193-6

[63] Bear MF, Malenka RC (1994) Synaptic plasticity: LTP and LTD. Curr Opin Neurobiol 4: 389–399. 7919934 10.1016/0959-4388(94)90101-5

[64] Chen KM, Yeh SL (2009) Asymmetric cross-modal effects in time perception. Acta Psychologica 130: 225–234. 10.1016/j.actpsy.2008.12.008 19195633

[65] Bausenhart K, Dolores de la Rosa M, Ulrich R (2013) Multimodal integration of time. Visual and auditory contributions to perceived duration and sensitivity. Experimental Psychology 18:1–13. 10.1027/1618-3169/a000249 24351985

[66] Burr DC, Banks MS, Morone MC (2009) Auditory dominance over vision in the perception of interval duration. Exp Brain Res 198:49–57. 10.1007/s00221-009-1933-z 19597804

[67] Miyazaki M, Nozaki D, Nakajima Y (2005) Testing Bayesian models of human coincidence timing. J. Neurophysiol 94: 395–399. 15716368 10.1152/jn.01168.2004

[68] Acerbi L, Wolpert D, Vijayakumar S (2012) Internal representations of temporal statistics and feedback calibrate motor-sensory interval timing. PLOS Computational Biology, 8, e1002771 10.1371/journal.pcbi.1002771 23209386 PMC3510049

